# Influence of ACE Gene I/D Polymorphism on Cardiometabolic Risk, Maximal Fat Oxidation, Cardiorespiratory Fitness, Diet and Physical Activity in Young Adults

**DOI:** 10.3390/ijerph18073443

**Published:** 2021-03-26

**Authors:** Adrián Montes-de-Oca-García, Alejandro Perez-Bey, Daniel Velázquez-Díaz, Juan Corral-Pérez, Edgardo Opazo-Díaz, María Rebollo-Ramos, Félix Gómez-Gallego, Magdalena Cuenca-García, Cristina Casals, Jesús G. Ponce-González

**Affiliations:** 1 MOVE-IT Research Group, Department of Physical Education, Faculty of Education Sciences, University of Cadiz, 11519 Puerto Real, Spain; adrianmontes96@hotmail.com (A.M.-d.-O.-G.); daniel.velazquez@uca.es (D.V.-D.); juan.corral@uca.es (J.C.-P.); ed.opazodiaz@alum.uca.es (E.O.-D.); maria.rebollo.ramos@gmail.com (M.R.-R.); jesusgustavo.ponce@uca.es (J.G.P.-G.); 2 Instituto de Investigación Biomédica de Cádiz (INiBICA), Hospital Uniersitario Puerta del Mar, Universidad de Cádiz, 11009 Cádiz, Spain; alejandro.perezperez@uca.es (A.P.-B.); magdalena.cuenca@uca.es (M.C.-G.); 3 GALENO Research Group, Department of Physical Education, Faculty of Education Sciences, University of Cadiz, 11519 Puerto Real, Spain; 4 Department of Physical Therapy, Faculty of Medicine, University of Chile, Independencia 1027, Santiago 8380453, Chile; 5 Faculty of Health Sciences, International University of La Rioja, 26006 Logroño, Spain; 2010gmz@gmail.com

**Keywords:** genetic association studies, heart diseases, lipid metabolism, healthy lifestyle, angiotensin-converting enzyme, obesity

## Abstract

There is controversy about the relationship between ACE I/D polymorphism and health. Seventy-four healthy adults (*n* = 28 women; 22.5 ± 4.2 years) participated in this cross-sectional study aimed at determining the influence of ACE I/D polymorphism, ascertained by polymerase chain reaction, on cardiometabolic risk (i.e., waist circumference, body fat, blood pressure (BP), glucose, triglycerides, and inflammatory markers), maximal fat oxidation (MFO), cardiorespiratory fitness (maximal oxygen uptake), physical activity and diet. Our results showed differences by ACE I/D polymorphism in systolic BP (DD: 116.4 ± 11.8 mmHg; ID: 116.7 ± 6.3 mmHg; II: 109.4 ± 12.3 mmHg, *p* = 0.035) and body fat (DD: 27.3 ± 10.8%; ID: 22.6 ± 9.7%; II: 19.3 ± 7.1%, *p* = 0.030). Interestingly, a genotype*sex interaction in relativized MFO by lean mass (*p* = 0.048) was found. The DD polymorphism had higher MFO values than ID/II polymorphisms in men (8.4 ± 3.0 vs. 6.5 ± 2.9 mg/kg/min), while the ID/II polymorphisms showed higher R-MFO values than DD polymorphism in women (6.6 ± 2.3 vs. 7.6 ± 2.6 mg/kg/min). In conclusion, ACE I/D polymorphism is apparently associated with adiposity and BP, where a protective effect can be attributed to the II genotype, but not with cardiorespiratory fitness, diet and physical activity. Moreover, our study highlighted that there is a sexual dimorphism in the influence of ACE I/D gene polymorphism on MFO.

## 1. Introduction

Cardiovascular diseases (CVD) are one of the main causes of mortality in developed countries [1], thus, cardiometabolic risk (CMR) factors should be analyzed in different populations, including hypertension, dietary risk, adiposity, hyperglycemia and low physical fitness [2]. Furthermore, it has been shown that inflammatory markers can be equal to or even more useful than traditional risk factors in predicting future cardiometabolic events [3]. Nonetheless, the study of the genetic component of these diseases is essential, since in most cases the origin of CVD is multifactorial. Thus, the study of heritage and its relationship with CMR factors could help clarify the causes of CVD attending to the influence of relevant factors including biological sex [4].

In this line, a human angiotensin I-converting enzyme (ACE) gene insertion/deletion (I/D) polymorphism, located on chromosome 17q23, is associated with CVD [5,6]. The ACE I/D polymorphism consists in the presence (insertion, I) or absence (deletion, D) of a 287 base pair fragment in intron 16 of ACE gene [7]. The ACE gene produces the protein ACE, which converts angiotensin (AngI) into angiotensin II (AngII), a potent vasoactive peptide that leads to effects mainly hypertensive, highlighting its role in the physiology of blood vessels and inflammation [5,8]. Indeed, higher plasma levels of ACE have been reported when the D allele was present [5], accordingly, the ACE DD genotype has been established as an independent risk factor of CVD and hypertension [6,9]. Similarly, a recent meta-analysis [10] has shown a higher risk of hypertrophic cardiomyopathy in the DD genotype than in the II genotype; therefore, a protective effect against CVD risk can be attributed to the II genotype.

Moreover, the cardiorespiratory fitness (CRF), expressed as maximal oxygen uptake (VO_2_max), has been established as a main preventive factor of CVD [1] and there seems to be a tendency for higher VO_2_max with the ACE II genotype [11], although with inconsistent associations in the literature [12]. Regarding the aerobic capacity and health, the maximal fat oxidation (MFO) capacity has been posed as a key indicator of metabolic flexibility [13] and, consequently, a lower CMR [14]. Nevertheless, only one study has investigated the influence of ACE I/D polymorphism on resting fat oxidation [15] and, to our best knowledge, there are no previous studies on the relationship between ACE gene I/D polymorphism and MFO despite its relevance to CVD and weight management.

The analysis of the genetic influence of the ACE I/D polymorphism on CMR and related outcomes should also consider age, physical activity and diet, among others [16]; moreover, sex differences can mediate the associations [4,16]. It should be note that most of the articles include a sample with a mean age over 55 years [10], therefore, the analysis in other ranges of age could be interesting.

Thus, the aims of the present study were (i) to determine the influence of ACE gene I/D polymorphism on CMR, MFO, CRF, diet and physical activity in young adults and (ii) to ascertain whether there are sex differences in these relationships.

## 2. Materials and Methods

### 2.1. Design

In this cross-sectional study from the “NutAF” project [14,17], the ACE gene I/D polymorphism was measured in a young adult population from Cadiz (Spain), and its possible influence with CMR and related outcomes was analyzed.

In this sense, CMR was determined considering waist circumference, body fat percentage, systolic (SBP) and diastolic (DBP) blood pressure, blood glucose, triglycerides, and inflammatory cytokines (TNF-α and IL-6); moreover, MFO and CRF (VO_2_max) were analyzed. All these measurements were performed in one day, in fasting conditions (>8 h), and in the morning (from 8:00 until 11:30 am). Additionally, the week before measurements, physical activity and diet were registered.

The study was approved by the Ethical Committee of the Hospital Puerta del Mar (Cadiz, Spain), in accordance with the Declaration of Helsinki. All tests were performed in the Laboratory of Physical Activity and Exercise of the University of Cadiz (Puerto Real, Spain).

### 2.2. Subjects

A total of 74 subjects (28 females) participated in the study and, apart from being obese, all subjects were apparently healthy but with different metabolic profiles. The characteristics of the participants are shown in Table 1. The inclusion criteria were: being between 18 and 45 years old, having a stable body mass (±2 kg) during the last 6 months and having a Body Mass Index (BMI) between 18.5 and 40 kg m^−2^. The exclusion criteria were: having made a weight-loss diet or a specific diet different to the regular one during the last 6 months or suffering any illness or injury that prevented physical exercise. Written informed consent was obtained by all participants after being informed about the nature of the study, its protocol and possible risks arising from the measurements.

### 2.3. Procedure

Initially, physical activity and diet were analyzed during the week before measurements, thus, participants were asked to maintain lifestyles during this period.

On the day of measurements, fasting blood samples and laboratory tests were performed. Participants were instructed to avoid intense physical activity the day before, to maintain their usual diet/hydration, and to avoid the intake of alcohol and caffeine the day before.

Firstly, heart rate and blood pressure were registered. Secondly, blood samples were taken in order to later determine the ACE gene I/D polymorphism, and those plasma markers used to estimate CMR, i.e., glucose, triglycerides, tumor necrosis factor-α (TNF-α), and interleukin-6 (IL-6). After that, anthropometric and body composition measurements were performed, then, a face mask was placed for the analysis of basal metabolism and the exercise protocol.

### 2.4. Physical Activity

The physical activity of the participants was objectively measured through accelerometry (GT3X+, Actigraph TM, LLC, Fort Walton Beach, FL, USA) for 7 consecutive days before the laboratory tests. The accelerometer was attached tightly in the hip, on the back side, with the notch faced upwards, and the participants were instructed to use it during waking hours and remove it during water-based activities. Only the participants wearing the accelerometers for ≥10 h/day during at least 4 days (including at least 1 weekend day) were included in the final analyses. Total physical activity was calculated to estimate energy balance according to the dietary intake. In addition, moderate to vigorous physical activity (MVPA) was calculated, considered any activity from moderate to very vigorous (over 2691 counts/min).

### 2.5. Dietary Assessment

Dietary intake was estimated through a 5-day dietary record the week before the laboratory tests, including three consecutive weekdays and both days of the weekend. All the participants were instructed to correctly fill it, recording each food by the weighing method. DIAL software (version 1.19) was used to estimate dietary energy intake. The energy balance was obtained subtracting the daily caloric expenditure (obtained from the sum of the basal metabolism and the caloric expenditure of physical activity) from the dietary energy intake. Likewise, dietary patterns were assessed concerning adherence to Mediterranean diet by using a previously validated questionnaire of 14 items [18].

### 2.6. Heart Rate and Blood Pressure Measurements

For the heart rate and blood pressure measurements, the participants were sitting in a chair, relaxed and with their feet on the floor. After 10 min, measurements of systolic blood pressure (SBP), diastolic blood pressure (DBP) in the non-dominant arm were taken with an Omron M3 digital monitor (HEM-7051-E, Kyoto, Japan). The average of three measurements, taken one minute apart, was used for analyses. Heart rate was continuously measured (at rest and during exercise) with a Polar Team 2 (Polar Electro Inc., Lake Success, NY, USA). Moreover, blood pressure recovery after exercise was taken into account using a third minute of recovery SBP to peak exercise SBP ratio [19].

### 2.7. Blood Extraction

Blood sample was taken from the antecubital vein, after 10 min in sitting position, by a nurse. Blood samples were collected in tubes containing EDTA and centrifuged to obtain plasma at 2500 rpm for 15 min at 4 °C. Clotted and hemolysed samples were discarded. Plasma was stored at −80 °C for subsequent analysis.

### 2.8. Genomic Typing of the ACE Gene I/D Polymorphism

Genomic Deoxyribonucleic acid (DNA) was extracted from peripheral blood anti-coagulated with EDTA using a standard phenol/chloroform procedure followed by alcohol precipitation. A polymerase chain reaction (PCR) amplification was performed using a StepOne™ Real-Time PCR System (Life Technologies, Foster City, CA, USA). Allelic discrimination analysis for the ACE gene I/D polymorphism was performed by PCR followed by electrophoresis on a 1.5% agarose gel containing ethidium bromide [20]. The primers used were: 5′-CTGGAGAGCCACTCCCATCCTTTCT and 5′-GACGTGGCCATCACATTCGTCAGAT. The amplified fragments were a 190 bp product for allele D (allele without insertion) and a 490 bp product for allele I (allele with insertion). In order to avoid the diagnosis of false negatives (ID heterozygotes misclassified as DD homozygotes), a second PCR reaction was performed in all samples initially classified as DD [21], with these insertion-specific primer pair: 5′-TGGGACCACAGCGCCCGCCACTAC and 5′-TCGCCAGCCCTCCCATGCCCATAA. Only the allele I produced a 335 bp fragment, identified on a 1.5% agarose gel stained with ethidium bromide performed in all of the samples initially classified as DD with these.

### 2.9. Plasma Biochemical Parameters

At the time of the analysis, samples were thawed on ice and then pipetted in duplicates of 10 µL into the microplates with 200 µL of the specific reagent from each commercial kit. In all plates, pattern curves of known concentrations of each parameter in question were added, in order to identify the absorbance data. For the analysis, the instructions of the manufacturer Spinreact (Spinreact SA, Sant Esteve d’en Bas, Girona, Spain) were followed and, by adapting the measurements to 96-well microplates, the metabolic parameters were analyzed in plasma including the levels of blood glucose (Glucose-HK Ref. 1001200) and triglycerides (TAG: Ref. 1001311). Subsequently, the microplates were introduced in a BIO-TEK PowerWaveTM 340 microplate reader and the absorbance readings were processed with the BIO-TEK KC JuniorTM program (Bio-Tek Instruments Inc., Winooski, VT, USA). Two inflammatory markers, TNF-α and IL-6, were determined by Bio-Plex 200 Systems with Bio-Plex ProTM Human Cytokine Assay kits (Bio-Rad Laboratories Inc., Shanghai, China).

### 2.10. Anthropometry and Body Composition

Height was measured in a standing position, after normal expiration, using a height rod (SECA 225, Hamburg, Germany; range of 60 to 200 cm, precision of 1 mm). Waist circumference was measured using a plastic anthropometric tape (SECA 201, Hamburg, Germany; range of 0 to 205 cm, precision of 1 mm) at the midpoint between the costal margin and iliac crest in the mid-axillary line in standing position at the end of a gentle expiration. The measurements were performed twice, and the average values were used for the analysis. Body mass (kg), body fat (kg and %) and lean mass (kg and %) were evaluated using a multi-frequency bioimpedance of 8 electrodes (TANITA-MC780MA, Barcelona, Spain). The subjects wore light clothing and adopted a specific posture according to the manufacturer’s instructions. All participants had to urinate before the test, which should be done without any metallic object in the body that could alter the results. BMI was determined as body mass (kg) divided by the square of height (m^−2^).

### 2.11. Basal Metabolism

Oxygen uptake (VO_2_) and carbon dioxide production (VCO_2_) were registered in resting conditions lying on a bed in a supine position for 30 min for calculating RER (Respiratory Exchange Ratio) and fat oxidation in a conditioned room (21 ± 1 °C, 50 ± 2% Relative Humidity). A mask was placed on the subject’s face to collect gas samples. An indirect circuit gas analyzer, Jaeger MasterScreen CPX^®^ (CareFusion, San Diego, CA, USA) was used to register indirect calorimetry data. Calibrations were daily performed before each measurement. During the test, the gas analyzer values were captured breath-by-breathe and averaged every 20 s. For the analysis of these variables, the first 5 min of the evaluation were eliminated and a stable period of 5 min was selected with a coefficient of variation for VO_2_ and VCO_2_ lower than 15%. The average values of VO_2_ and VCO_2_, in the selected time interval, were used to calculate the basal metabolism (kcal) by an indirect equation proposed by Frayn [22].

### 2.12. Maximal Fat Oxidation (MFO) and Cardiorespiratory Fitness (VO_2_max)

An incremental protocol in cycle ergometer (Lode Excalibur, Groningen, The Netherlands) was designed from the standardized protocol [23], with two consecutive phases to determine MFO and maximal oxygen uptake (VO_2_max). The first phase, for the determination of MFO, consisted of 3-min steps with 15 W increments in overweight/obese subjects and 30 W in subjects with normal weight, with a maintained pedaling rate between 60–80 rpm. This phase was interrupted when RER ≥ 1. After a brief pause (between 3 and 5 min), the second phase to detect VO_2_max was initiated. This phase began at the load at which phase 1 ended, and continued with 1-min steps increasing at the same load rate as in phase 1, with equal cadence. This phase ended when the participant reached exhaustion. The protocol was considered maximum when the VO_2_ reached a plateau, the theoretical maximal heart rate was reached and when RER ≥ 1.10. When the aforementioned maximality criteria were not met, the VO_2_peak was used. RER, VO_2_ and VCO_2_ were measured by indirect calorimetry (Jaeger MasterScreen CPX^®^). To calculate the fat oxidation in the different steps of the protocol, the average values of VO_2_ and VCO_2_ were used in the last 60 s of each step of the test, applying the Frayn equation [22]. Similarly, the average value of VO_2_ was used to determine the % VO_2_max reached in each step. With the values obtained from fat oxidation and % VO_2_max in each step, a polynomial curve that best fits the results of the present analysis were drawn for each participant.

### 2.13. Statistical Analysis

Chi-squared (χ^2^) test was used to verify the Hardy-Weinberg equilibrium and to describe both the genotypic and allelic frequencies. Normality of distribution and homogeneity of variance were contrasted by applying the Kolmogorov-Smirnov and Levene tests.

Descriptive statistics were calculated and expressed as mean ± standard deviation (SD), comparison between sexes were determined through a Student *t* test. All outcomes were separately analyzed, additionally, two CMR clusters were calculated. A CMR cluster according to sex was created by using standardized values (Z-score) [(value − mean)/standard deviation] of waist circumference, body fat percentage, SBP, DBP, blood glucose and triglycerides. Furthermore, a second CMR cluster was created including the inflammatory cytokines TNF-α and IL-6 to the aforementioned cluster model.

The differences between the groups according to the ACE gene I/D polymorphism (DD, ID and II) in CMR, MFO, CRF, physical activity and diet were analyzed by applying a 1-way analysis of variance (ANOVA) followed by the Bonferroni post-hoc comparisons. Moreover, a recessive model with two groups (DD vs. ID/II) was established and a Student *t* test was used to determine differences between groups.

The possible interaction of sex with the ACE gene I/D polymorphism in the analyzed variables was investigated through a 2-way ANOVA, followed by the Bonferroni post-hoc comparisons.

Effect size statistics were calculated, with Cohen’s d for Student *t* test, η^2^ for 1-way ANOVA, and η_p_^2^ for 2-way ANOVA. The level of significance was set at *p* ≤ 0.05. IBM SPSS Statistics 22 software (SPSS Inc., Chicago, IL, USA) was used for the statistical analysis.

## 3. Results

The Hardy-Weinberg equilibrium (HWE) test showed that χ^2^ = 0.801, *p* = 0.371, suggesting that the population is consistent with HWE, and confirming that the allele types were randomly sampled. The expected frequencies were DD (*p* = 0.2849) *n* = 21.1, ID (*p* = 0.4977) *n* = 36.8, and II (*p* = 0.2176) *n* = 16.1. The distribution of ACE genotype frequencies was 31% for DD, 45% for ID, and 24% for II.

Regarding descriptive characteristics of the participants (Table 1), as it was expected, men had higher height, body mass and lean body mass, with lower body fat percentage, than women. Moreover, men presented significantly higher SBP, with a trend in DBP (*p* < 0.10), higher blood glucose and TNF-α, compared with women. Men reported higher values in VO_2_max relativized to body mass and absolute MFO than women; however, when VO_2_max and MFO were relativized to lean body mass showed no differences between sexes.

The differences between ACE gene variants (DD, ID and II) on body composition, physical activity, diet, biochemical parameters, CRF, MFO and CMR of the participants are presented in Table 2. The II group presented lower body fat percentage than the DD group and lower SBP than DD and ID groups. In Table 3, the differences according to the recesive model of alleles (DD vs. ID/II) are presented, where the DD genotype showed higher body fat than the ID/II genotype, also with a similar trend in BMI and the CMR cluster with inflammatory cytokines.

**Figure 1:**
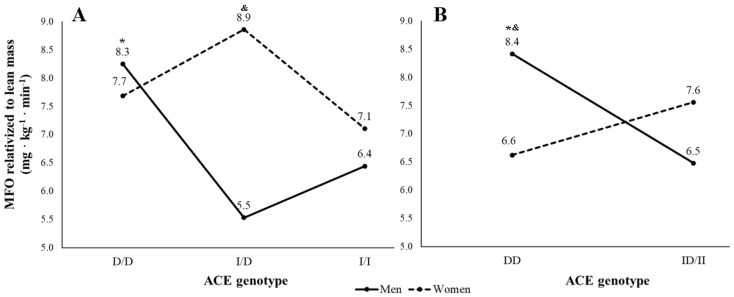
Interaction of sex and genotype of Angiotensin-converting enzyme (ACE) gene I/D polymorphism in Maximal Fat Oxidation (MFO) relativized to lean mass. Panel (**A**) shows the analysis considering the three ACE gene variants (DD, ID and II) after accounting by body fat percentage. Panel (**B**) shows the analysis according to the recessive model of ACE gene (DD vs. ID/II). * *p* ≤ 0.05 vs. other genotype groups. & *p* ≤ 0.05 vs. women of the same genotype group. Sample size was 11 and 12 men and women respectively in DD, and 35 and 16 men and women respectively in ID/II in Panel (**A**,**B**).

One of the main aims of this study was to analyze the sex and genotype interaction. In this sense, there was a sex × genotype interaction in R-MFO with body fat percentage as covariable (F(2,66) = 3.35; *p* = 0.041; η_p_^2^ = 0.092) (Figure 1A). Similarly, attending the recessive model (DD vs. ID/II), a significant interaction between sex and genotype was found in absolute MFO (F(2,69) = 3.10; *p* = 0.049; η_p_^2^ = 0.054) and R-MFO (F(2,69) = 3.90; *p* = 0.048; η_p_^2^ = 0.065). Specifically, the DD polymorphism had higher R-MFO values than ID/II polymorphisms in men (8.4 ± 3.0 vs. 6.5 ± 2.9 mg/kg/min), while the ID/II polymorphisms showed higher R-MFO values than DD polymorphism in women (6.6 ± 2.3 vs. 7.6 ± 2.6 mg/kg/min) (Figure 1B). The sex × genotype analyses did not show other statistically significant interactions in the registered variables.

## 4. Discussion

Our results highlighted that some CMR-related outcomes presented differences according to the ACE I/D gene polymorphism in young adults. Similarly to previous research [6], in our participants the II genotype presented lower body fat percentage and lower SBP than DD and ID genotypes. Accordingly, previous studies have found associations between ACE gene I/D polymorphism and CMR, where the DD polymorphism presented higher metabolic disturbances than ID/II polymorphisms partly due to a greater activity level of ACE protein [24,25]. Therefore, those subjects with II genotype seem to have a lower CMR and lower cardiovascular events than DD genotype [10]. These results support that the ACE gene I/D polymorphism partly influences cardiometabolic health and adiposity, although it should not be forgotten that genetics does not fully determine the phenotype of individuals, since environmental factors also play an important role.

Regarding the study of relevant factors in modulating the relationship of genotype and health outcomes, it becomes important the analysis of sex differences. In this line, the main finding of the present study was that there is a sexual dimorphism in the influence of ACE I/D gene polymorphism on MFO. Additionally, it has been previously reported a sex-specific effect of ACE gene I/D polymorphism on other CMR parameters, such as blood pressure and hypertension [26,27]; however, our study only reported significant sex*genotype interactions in MFO, the main variable regarding metabolic flexibility.

Under our knowledge, this is the first study that determines the influence of ACE gene I/D polymorphism on MFO. Specifically, our study described that MFO was higher in DD genotype compared with ID/II genotype for men, while it was higher in ID/II genotype compared with DD genotype for women. Therefore, the influence of the ACE I/D gene polymorphism on cardiometabolic flexibility and the ability to oxidize fats during exercise may be sex-dependent, and seems to be independent of CRF (VO_2_max). According to a previous study [28], which analyzed the influence of androgen receptor CAG and GGN repeat polymorphisms on MFO, the mentioned sexual dimorphism could be explained by hormonal differences between men and women; however, the study was performed only in young males, highlighting the interest of the present study.

The mechanisms that can explain these sex differences have not been fully established. However, it has been previously shown that estrogens may protect female rats against hypertension by amplifying the vasodilator contributions of Ang-(1–7) and reducing formation of AngII [29,30,31]. Higher levels of Ang-I and Ang-II were found in DD females in a cohort of healthy individuals [32]. Ang-II hormone stimulates vasoconstriction which could reduce the arrival of oxygen and fatty acids from adipose tissue to the skeletal muscle. In fact, it has been reported that high levels of Ang-II reduce the sympatholysis observed during physical exercise, which could explain lower fat oxidation in women belonging to the DD genotype of our study.

These results could be applied to healthy lifestyle interventions aimed at preventing or reducing overweight and obesity. In this line, most interventions are focused on increasing physical activity or on reducing calorie intake through diet or on increasing physical activity and exercise. However, the adherence to these interventions sometimes is scarce. Thus, the possible differences between genotypes could help to individually adjust physical activity and dietary programs.

Regarding physical activity and exercise, a review concluded that individuals with at least one ACE D allele had significantly better functional performance after exposure to higher levels of physical activity or after an exercise intervention than those homozygous for ACE I allele [33]. However, other studies have not found differences of physical activity levels according to the ACE I/D gene polymorphism, similarly to our findings [34]. Thus, currently there is no clear consensus on whether the insertion (I) polymorphism or the deletion (D) polymorphism could affect cardiometabolic flexibility and aerobic capacity. Some reviews [35,36] have concluded that the limited number of genetic variants studied in small and heterogeneous cohorts is a common limitation in the field of exercise genomics. Moreover, these discrepancies can be due to the fact that there is a sexual dimorphism in the influence of ACE I/D gene polymorphism on MFO. Therefore, future interventional studies should consider this aspect to elucidate the ACE-polymorphism specific responses to physical exercise.

Related to dietary interventions, interestingly, a previous study in obese women [15] showed a higher reduction of body fat in the II/ID genotype compared with the DD genotype after a calorie-restricted dietary intervention with statistically significant differences in resting fat oxidation. However, in our study, caloric energy balance and dietary patterns (adherence to Mediterranean diet) was not determined by the ACE I/D gene polymorphism. It should be noted that no previous studies have investigated the influence of ACE gene I/D polymorphism on energy balance and adherence to Mediterranean diet. Therefore, the present study offers novel information indicating that dietary patterns are probably largely determined by environmental factors in young adults; however, as it was mentioned before, adiposity differed between groups.

This study is not exempt from critics and limitations. Firstly, the sample size of this cross-sectional study is scarce to raise conclusions (e.g., there are only 6 women in the II group). Secondly, the population is young and, despite obesity, healthy; therefore, extrapolation of results should take this into account. Future studies including a higher sample with greater heterogeneity in CMR, physical activity and CRF parameters and longitudinal designs are warranted. Finally, the measurement of body composition by bioimpedance instead of using another more precise method like Dual Energy X-ray Absorptiometry (DXA) is also a limitation. Notwithstanding, the determination of MFO and CRF in a controlled situation (with a laboratory test, gas analyzer, etc.) and the number of registered outcomes bring strengths to the design.

## 5. Conclusions

In conclusion, ACE I/D polymorphism is apparently associated with higher adiposity and BP, but not with CRF, diet and physical activity. Moreover, our study highlighted that there is a sexual dimorphism in the influence of ACE I/D gene polymorphism on R-MFO. This fat oxidation capacity was higher in DD genotype than in II/ID genotype in men; while R-MFO was higher in II/ID genotype than in DD one in women. Therefore, physical exercise interventions that are performed at this intensity (low-intensity aerobic training) can have different responses in a young adult population according to the ACE I/D polymorphism and sex. Nevertheless, more studies are needed in order to deepen in the research of ACE gene I/D polymorphism and its relationship with health.

## Figures and Tables

**Table 1:** General characteristics of total sample and differences between men and women.

	Total			Men			Women			*p*	*d*
Age (years)	22.46	±	4.21	22.23	±	3.59	22.88	±	5.21	0.526	−0.15
Height (cm)	172.06	±	8.85	176.68	±	6.32	163.52	±	6.07	**<0.001**	**2.11**
Body Mass (kg)	74.97	±	15.74	77.84	±	14.43	69.69	±	16.95	**0.033**	**0.53**
Lean body mass (kg)	54.38	±	8.92	59.26	±	6.41	45.37	±	5.00	**<0.001**	**2.35**
Body fat (%)	21.97	±	9.34	18.28	±	7.08	28.77	±	9.30	**<0.001**	**−1.31**
Body Mass Index (kg · m^−2^)	25.36	±	5.48	24.87	±	3.87	26.28	±	7.64	0.292	−0.25
Waist circumference (cm)	82.27	±	14.33	83.73	±	12.19	79.92	±	17.24	0.289	0.27
Total MVPA (min/week)	501.96	±	166.99	480.95	±	146.25	539.14	±	196.08	0.157	−0.35
Energy balance (kcal/day)	280.26	±	1008.83	303.99	±	1164.61	236.45	±	647.19	0.785	0.07
MD adherence (0–14 range)	6.99	±	1.78	7.21	±	1.92	6.58	±	1.45	0.146	0.36
Systolic Blood Pressure (mmHg)	114.20	±	10.26	116.70	±	7.88	109.52	±	12.56	**0.004**	**0.73**
Diastolic Blood Pressure (mmHg)	68.46	±	9.88	66.96	±	9.00	71.27	±	10.98	0.078	−0.44
Blood pressure recovery (ratio)	0.82	±	0.30	0.79	±	0.34	0.88	±	0.19	0.242	−0.29
Plasma glucose (mg/dL)	101.76	±	10.02	103.90	±	9.55	97.83	±	9.84	**0.016**	**0.63**
Plasma triglycerides (mg/dL)	69.06	±	24.83	70.28	±	24.52	66.84	±	25.77	0.589	0.14
Tumor necrosis factor-α (pg/mL)	8467.15	±	7574.43	9934.35	±	8443.06	5960.69	±	5027.59	**0.040**	**0.54**
Interleukin-6 (pg/mL)	473.44	±	338.41	504.84	±	370.25	408.79	±	259.09	0.342	0.29
BM VO_2_max (mL · kg^−1^ · min^−1^)	42.10	±	11.49	46.07	±	10.11	34.93	±	10.42	**<0.001**	**1.09**
LM VO_2_max (mL · kg^−1^ · min^−1^)	18.90	±	4.54	18.80	±	4.84	19.09	±	4.00	0.789	−0.07
Absolute MFO (g · min^−1^)	0.38	±	0.15	0.40	±	0.17	0.32	±	0.11	**0.029**	**0.55**
R-MFO (mg ·kg^−1^ · min^−1^)	7.03	±	2.86	6.95	±	3.05	7.16	±	2.57	0.768	−0.07
Clustered CMR (Z-score)	0.32	±	3.29	0.50	±	3.15	0.04	±	3.56	0.605	0.14
INFLAM-Clustered CMR (Z-score)	−0.03	±	3.14	0.10	±	3.39	−0.24	±	2.79	0.732	0.11
N	74			46			28				

Sex differences (*p* < 0.05) in the Student *t* test appear in bold. Values are expressed as mean ± standard deviation. Abbreviations: d, Cohen’s d; MVPA, Moderate to vigorous physical activity; MD, Mediterranean diet; BM, Body mass; LM, Lean mass; VO_2_max, Maximal oxygen uptake; R-MFO, Maximal fat oxidation rate relativized to lean mass; CMR, Cardiometabolic risk; INFLAM-Clustered CMR, Cluster of CMR which includes TNF-α and IL-6.

**Table 2:** Differences in the analyzed outcomes between the three ACE gene I/D polymorphism groups.

	DD			ID			II			*p*		η^2^
Number of men/women	11/12			23/10			12/6			-		-
Age (years)	23.57	±	5.29	22.33	±	4.29	21.83	±	2.07	0.390		0.03
Height (cm)	170.67	±	10.32	173.09	±	8.31	172.34	±	7.12	0.593		0.01
Body Mass (kg)	79.71	±	17.44	76.94	±	16.31	70.77	±	11.81	0.194		0.05
Lean body mass (kg)	53.66	±	8.20	55.37	±	9.80	53.51	±	7.90	0.694		0.01
Body fat (%)	27.29	±	10.82	22.55	±	9.72	19.31	±	7.13	**0.030**	*	**0.09**
Body Mass Index (kg · m^−2^)	27.64	±	7.38	25.63	±	4.89	23.78	±	3.33	0.088		0.07
Waist circumference (cm)	86.78	±	16.89	84.01	±	14.81	78.26	±	9.77	0.223		0.05
Total MVPA (min/week)	501.55	±	171.02	469.59	±	175.24	562.75	±	131.27	0.164		0.05
Energy balance (kcal/day)	159.89	±	854.92	529.75	±	1290.38	9.12	±	652.53	0.187		0.05
MD adherence (0–14 range)	6.83	±	1.75	6.76	±	1.66	7.41	±	2.00	0.442		0.02
Systolic Blood Pressure (mmHg)	116.38	±	11.84	116.72	±	6.25	109.44	±	12.32	**0.035**	*&	**0.09**
Diastolic Blood Pressure (mmHg)	70.68	±	11.12	68.89	±	9.26	66.85	±	8.87	0.466		0.02
Blood pressure recovery (ratio)	0.88	±	0.20	0.83	±	0.27	0.83	±	0.31	0.731		0.01
Plasma glucose (mg/dL)	98.68	±	11.61	101.56	±	9.57	103.00	±	9.25	0.376		0.03
Plasma triglycerides (mg/dL)	67.20	±	23.09	71.36	±	26.29	70.54	±	24.94	0.822		0.01
Tumor necrosis factor-α (pg/mL)	8691.86	±	9769.21	6489.78	±	5639.46	11,644.02	±	6755.80	0.083		0.07
Interleukin-6 (pg/mL)	609.51	±	495.12	422.08	±	292.73	428.72	±	165.75	0.198		0.06
BM VO_2_max (mL · kg^−1^ · min^−1^)	37.30	±	13.15	42.25	±	11.93	43.46	±	9.40	0.189		0.05
LM VO_2_max (mL · kg^−1^ · min^−1^)	18.05	±	4.04	18.57	±	5.27	19.47	±	3.73	0.617		0.01
Absolute MFO (g · min^−1^)	0.40	±	0.17	0.35	±	0.15	0.38	±	0.16	0.493		0.02
R-MFO (mg ·kg^−1^ · min^−1^)	7.48	±	2.80	6.66	±	3.04	7.11	±	2.66	0.582		0.02
Clustered CMR (Z-score)	0.89	±	3.64	0.73	±	3.47	−0.65	±	2.09	0.323		0.03
INFLAM-Clustered CMR (Z-score)	1.35	±	3.01	−0.28	±	3.54	−0.74	±	1.80	0.172		0.08
N	23			33			18					

Sex differences (*p* < 0.05) in the 1-way ANOVA appear in bold. * means significant differences between II and DD and ^&^ between II and ID in the Bonferroni post-hoc comparison. Values are expressed as mean ± standard deviation. Abbreviations: d, Cohen’s d; MVPA, Moderate to vigorous physical activity; MD, Mediterranean diet; BM, Body mass; LM, Lean mass; VO_2_max, Maximal oxygen uptake; R-MFO, Maximal fat oxidation rate relativized to lean mass; CMR, Cardiometabolic risk; INFLAM-Clustered CMR, Cluster of CMR which includes TNF-α and IL-6.

**Table 3:** Comparisons of the recesive model (DD vs. ID/II) in the analysed outcomes.

	DD			ID/II			*p*	*d*
Number of men/women	11/12			35/16			*-*	*-*
Age (years)	23.57	±	5.29	22.16	±	3.65	0.188	0.33
Height (cm)	170.67	±	10.32	172.83	±	7.85	0.326	−0.25
Body Mass (kg)	79.71	±	17.44	74.76	±	15.05	0.217	0.31
Lean body mass (kg)	53.66	±	8.20	54.71	±	9.13	0.638	−0.12
Body fat (%)	27.29	±	10.82	21.41	±	8.96	**0.017**	**0.61**
Body Mass Index (kg · m^−2^)	27.64	±	7.38	24.98	±	4.46	0.058	0.48
Waist circumference (cm)	86.78	±	16.89	82.13	±	13.54	0.226	0.32
Total MVPA (min/week)	501.55	±	171.02	503.13	±	165.67	0.971	−0.01
Energy balance (kcal/day)	159.89	±	854.92	346.00	±	1128.52	0.484	−0.18
MD adherence (0–14 range)	6.83	±	1.75	6.98	±	1.79	0.732	−0.09
Systolic Blood Pressure (mmHg)	116.38	±	11.84	114.10	±	9.47	0.382	0.22
Diastolic Blood Pressure (mmHg)	70.68	±	11.12	68.15	±	9.09	0.308	0.26
Blood pressure recovery (ratio)	0.88	±	0.20	0.83	±	0.28	0.427	0.20
Plasma glucose (mg/dL)	98.68	±	11.61	102.07	±	9.39	0.187	−0.33
Plasma triglycerides (mg/dL)	67.20	±	23.09	71.07	±	25.57	0.537	−0.16
Tumor necrosis factor-α (pg/mL)	8691.86	±	9769.21	8207.86	±	6447.65	0.804	0.06
Interleukin-6 (pg/mL)	609.51	±	495.12	424.63	±	249.00	0.071	0.54
BM VO_2_max (mL · kg^−1^ · min^−1^)	37.30	±	13.15	42.69	±	11.01	0.072	−0.46
LM VO_2_max (mL · kg^−1^ · min^−1^)	18.05	±	4.04	18.89	±	4.76	0.468	−0.18
Absolute MFO (g · min^−1^)	0.40	±	0.17	0.36	±	0.15	0.337	0.24
R-MFO (mg ·kg^−1^ · min^−1^)	7.48	±	2.80	6.83	±	2.89	0.367	0.32
Clustered CMR (Z-score)	0.89	±	3.64	0.27	±	3.12	0.469	0.19
INFLAM-Clustered CMR (Z-score)	1.35	±	3.01	−0.43	±	3.03	0.066	0.59
N	23			51				

Sex differences (*p* < 0.05) in the Student *t* test appear in bold. Values are expressed as mean ± standard deviation. Abbreviations: d, Cohen’s d; MVPA, Moderate to vigorous physical activity; MD, Mediterranean diet; BM, Body mass; LM, Lean mass; VO_2_max, Maximal oxygen uptake; R-MFO, Maximal fat oxidation rate relativized to lean mass; CMR, Cardiometabolic risk; INFLAM-Clustered CMR, Cluster of CMR which includes TNF-α and IL-6.

## Data Availability

NutAf Project. Financed by Universidad de Cádiz.
